# Reducing kidney motion: optimizing anesthesia and combining respiratory support for retrograde intrarenal surgery: a pilot study

**DOI:** 10.1186/s12894-019-0491-3

**Published:** 2019-07-05

**Authors:** Nariman Gadzhiev, Ullubiy Oibolatov, Leonid Kolotilov, Sergei Parvanyan, Gagik Akopyan, Sergei Petrov, Courtney M. Cottone, John Sung, Zhamshid Okhunov

**Affiliations:** 1 Department of Urology, Pavlov First Saint Petersburg State Medical University, Lva Tolstogo 17, Saint-Petersburg, Russian Federation 197342; 2 Department of Anesthesiology, The Nikiforov Center of Emergency Medicine, Optikov 54, Saint-Petersburg, Russian Federation 197448; 3 Department of Urology, Sechenov First Moscow State Medical University, Optikov 54, Saint-Petersburg, Russian Federation 197448; 4 Department of Urology, University of California, Irvine, 333 City Boulevard West, Orange, CA 92868 USA

**Keywords:** General anesthesia (GA), Retrograde intrarenal surgery (RIRS), Mechanical ventilation (MV), Periodic apnea (PA), High frequency jet ventilation (HFJV), Shockwave lithotripsy (SWL), Urolithiasis, Nephrolithiasis, Combined respiratory support (CRS)

## Abstract

**Background:**

One of the greatest challenges presented with RIRS is the potential for movement of the stone within the operative field associated with diaphragm and chest respiratory excursions due to mechanical ventilation. To overcome this challenge, we propose in this pilot study a new general anesthesia technique combining high frequency jet ventilation (HFJV) with small volume mechanical ventilation (SVMV). Data regarding safety, feasibility and surgeons’ impression was assessed.

**Methods:**

Patients undergoing RIRS for kidney stones from November 2017 to May 2018 were prospectively recruited to participate in the study. In each case after the beginning of general anesthesia (GA) with mechanical ventilation (MV) surgeons were asked to assess the mobility of the operative field and conditions for laser lithotripsy according to the developed questionnaire scale. The questionnaire consisted of 5 degrees of assessment of kidney mobility and each question was scored from 1 to 5, 1 being very mobile (extremely poor conditions for dusting) and 5 completely immobile (Ideal conditions for dusting).

After the assessment GA was modified with combined respiratory support (CRS), reducing tidal volume and respiratory rate (small volume mechanical ventilation, SVMV) and applying in the same time transcatheter high frequency jet ventilation (HFJV) inside the closed circuit.

After beginning of CRS, surgeons were once again asked to assess the mobility of the operative field and the conditions for laser lithotripsy. Main ventilation parameters were recorded and compared in both regimens.

**Results:**

A total of 38 patients were included in the study. The mean age was 49 (range 45–53) with a mean stone size of 10 mm (range 10–14) and Hounsfield unit of 1060 (range 930–1190). All patients underwent successful RIRS and no intraoperative complications occurred throughout the duration of the study. A statistically significant difference between ventilation parameters prior to and after CRS institution was detected in all cases, however their clinical impact was negligible. Despite this, assessment via the questionnaire scale point values varied significantly before and after the application of CRS and were 2.3 (2.1; 2.6) and 3.8 (3.7; 4.0) respectively (*p* < 0.001).

**Conclusions:**

The novel combined respiratory approach consisting of HFJV and SVMV appears to provide better conditions for stone dusting through reduced respiratory kidney motion and is not associated with adverse health effects or complications.

**Trial registration:**

NCT03999255, date of registration: 25th June 2019 (retrospectively registered).

## Background

According to the currently established guidelines released by the European Association of Urology, retrograde intrarenal surgery (RIRS) via flexible ureterorenoscopy, is considered a well-established and effective procedure for the treatment of urolithiasis [1]. RIRS is most typically performed under general anesthesia (GA) with mechanical ventilation (MV) assistance, though can also be conducted under spinal or epidural anesthesia [2].

Regardless of anesthesia method one of the greatest challenges presented with RIRS is the potential for movement of the stone within the operative field associated with diaphragm and chest respiratory excursions due to MV. To overcome this impediment, Emiliani and Traxer suggested a technique known as periodic apnea (PA) to allow for momentary respites in respirations to facilitate stone removal and to minimize stone movements during laser lithotripsy [3]. However, surgeons may hesitate to induce PA due to relevant concerns of inducing hypercapnea. To circumvent these concerns, high frequency jet ventilation (HFJV) during GA has been suggested [4]. However, this method makes it impossible to use inhalational anesthetics and does not monitor end-tidal CO_2_ levels nor the volume of exhaled air [5].

Thus, to provide stability of the operative field during RIRS, we propose a modified technique of GA, referred to as combined respiratory support (CRS). It implies HFJV with small volume mechanical ventilation (SVMV). The purpose of this study is to perform a pilot study to assess the safety and feasibility of this technique during RIRS.

## Material and methods

After obtaining approval by the local ethics committee, we performed a prospective, single**-**center study in patients undergoing RIRS for renal stones from November of 2017 to May of 2018. Patients with kidneys stones with indications for RIRS, who were eligible to enroll in this study, were identified by the appropriate research personnel via diagnosis at an outpatient clinic visit. All patients provided written consent to participate in the study. Patients consented to publish the data obtained during the study. Thorough explanation of the study was provided to patients, after which all provided signed, informed consent. Patients with ASA class of greater than 3 and active urinary tract infection were excluded from the study. All procedures were performed by two experienced endourologists [6].

### Questionnaire

The secondary outcome of the study was to implement **a** subjective questionnaire in order to assess surgeon’s feedback on novel technique. The questionnaire consisted of 5 degrees of assessment of kidney mobility and each question was scored from 1 to 5, 1 being very mobile (extremely poor conditions for dusting), 2 being significantly mobile (unsatisfactory conditions for dusting), 3 being slightly mobile (satisfactory conditions for dusting), 4 almost immobile (good conditions for dusting) and 5 completely immobile (Ideal conditions for dusting) (Table 1). This was done in 2-step fashion in every patient and each patient served his own control: first, after the beginning of GA with MV in the mode of normal ventilation the questionnaire was implemented and surgeons were asked to assess the mobility of the operative field and the decency of conditions for laser lithotripsy. In addition to this assessment, anesthesia parameters including results of arterial blood gas analysis were recorded and evaluated in order to address safety concerns.

**Table 1:** Developed questionnaire scale assessing the mobility and conditions of the operative field

Classification	Description	Value
Very Mobile	Extremely poor conditions for dusting	1 point
Significantly Mobile	Unsatisfactory conditions for dusting	2 points
Slightly Mobile	Satisfactory conditions for dusting	3 points
Almost Immobile	Good conditions for dusting	4 points
Completely Immobile	Excellent conditions for dusting	5 points

Second, novel CRS technique was then instituted and maintained throughout RIRS. Before lithotripsy itself surgeons were once again asked to assess the mobility of the operative field and conditions for laser lithotripsy according to the previously mentioned questionnaire. Monitoring of end-tidal carbon dioxide (EtCO2) level was carried out by intermittent capnography. Besides main anesthesia parameters with results of arterial blood gas analysis were recorded at the end of lithotripsy while on CRS for later comparison.

### CRS technique

The concentration of Sevoflurane was increased from 1.4–2.6 vol% to 8 vol%. The following changes were then made to alter ventilation patterns: the tidal volume (Vt) (6–8 ml/kg IBW) and the respiratory rate (8–15 per minute) (RR) were decreased by 2–3 times, the ratio of inhalation to exhalation (I:E) was increased from 1:2 to 1:3, the fraction of inspired oxygen (FiO_2_) was decreased from 40 to 21% in the fresh gas flow (FGF) of 1.0 l/min. Transcatheter HFJV through tracheal tube was initiated with a ZisLINEJV-100 high frequency respirator (Triton-Electronics, Russia) with a respiratory cycle frequency (RCF) of 300 per minute, I:E = 1:3, FiO_2_ = 1.0 and working pressure (WP) 0.3–0.6 bar. Depth of anesthesia was measured by BIS Monitor (Covidien AG, Zurich, Switzerland).

### Surgical technique

RIRS was performed with the patient in the lithotomy position with 7.5 Flex X^2^ ureteroscope (Karl Storz, Tuttlingen, Germany). Lithotripsy was performed with a holmium laser using 270 μm fibers at 0.3–0.5 J and 20–40 Hz (VersaPulse® PowerSuite™ 100 W) with patient under CRS. Dusting technique was utilized in all patients. A fragment was deliberately left to be extracted for stone analysis. Ureteral access sheath was used in 2 cases (stone size 14 mm).

### Statistical analysis

Nonparametric resampling, permutation and randomization procedures (bootstrap and Monte Carlo) were applied for the statistical data analysis using software PAST [7]. Statistical significance of the observed effects was tested by *p-*values and confidence intervals (CIs). Threshold of 0.001 was considered as a critical significance level. For the expression of the clinical importance of findings, and their unification the so-called “standardized effect size” was used.

## Results

A total of 38 patients were enrolled in the study with a mean age of 49 years (range 45–53). Mean body mass index was 27 kg/m^2^ (range 25–28). Mean ASA score was 2 (range 1–3). Mean stone size was 10 mm (range 10–14). Mean stone density was 1060 (range 930–1190). All cases were successful. Mean duration of RIRS was 26 min (range 20–32). Stone free rate in the study confirmed by ultrasound and KUB at 8 weeks was 86% (Table 2).

**Table 2:** Patient and stone characteristics

Feature	Mean (range)
Sample size, *n*	38
Age, years	49 (45; 53)
Body mass index, kg/m2	27 (25; 28)
ASA, scores	2 (1; 3)
Maximum diameter of the stone, mm	10 (10; 14)
Density of the stone, HU	1060 (930; 1190)
Stone location	
Upper calyx	16
Middle calyx	11
Lower calyx	7
Pyelus	4
Duration of RIRS, min	26 (14; 32)
Stone free rate	86%

A statistically significant difference between ventilation parameters prior and after CRS institution was detected in all cases (Table 3). End-tidal CO_2_ with CRS implementation was _40_ 42 _43_ mmHg during RIRS which is in the range of normocapnia. No intraoperative complications occurred for any patients throughout the duration of the study. There was statistical significance in surgeons’ subjective questionnaire assessment points before and after the application of CRS 2.3 (2.1; 2.6) and 3.8 (3.7; 4.0) *p* < 0.0001, respectively (Table 2).

**Table 3:** Ventilation parameters and points without and with CRS technique

Features	Mean (95% *CI*)		Mean difference (*d* = *M*a – *M*b) (95% *CI*)	*p*-value
	*Without CRS* (*M*b)	*With CRS* (*M*a)		
V_E_ (l/min)	6.1 (5.8; 6.5)	9.3 (8.9; 9.7)	3.2 (2.6; 3.7)	< 0.001
P_peak_ (cm H_2_O)	18.2 (17.5; 18.8)	19 (18; 20)	0.7 (0.4; 1.0)	< 0.001
P_mean_ (cm H_2_O)	7.9 (7.7; 8.1)	6.7 (6.5; 6.9)	−1.2 (−1.4; −1.0)	< 0.001
SpO_2_ (%)	98 (97; 99)	99 (98; 100)	1.0 (0.2; 1.5)	< 0.001
EtCO_2_ (mm Hg)	36 (35; 37)	42 (40; 43)	6 (4; 7)	< 0.001
PaO_2_ (mm Hg)	180 (170; 190)	320 (300; 340)	170 (160; 190)	< 0.001
PaCO_2_ (mm Hg)	39 (38; 40)	43 (41; 45)	4.0 (0.4; 7.6)	< 0.001
Fi_Sev_ (vol%)	2.2 (2.1; 2.3)	7.3 (7.0; 7.6)	5.1 (4.9; 5.3)	< 0.001
Et_Sev_ (vol%)	1.8 (1.7; 2.0)	1.0 (0.9; 1.1)	−0.8 (−0.9; −0.7)	< 0.001
MAC	0.91 (0.87; 0.95)	0.52 (0.48; 0.56)	−0.39 (−0.44; −0.34)	< 0.001
BIS	44 (42; 47)	53 (49; 56)	9 (5; 13)	< 0.001
Points	2.3 (2.1; 2.6)	3.8 (3.7; 4.0)	1.6 (1.3; 1.8)	< 0.001

Notations: C*I* – confidence intervals; Mb and *Ma* – mean values *before and after the application of the CRS technique*, respectively; *d* – mean difference

**Fig. 1:**
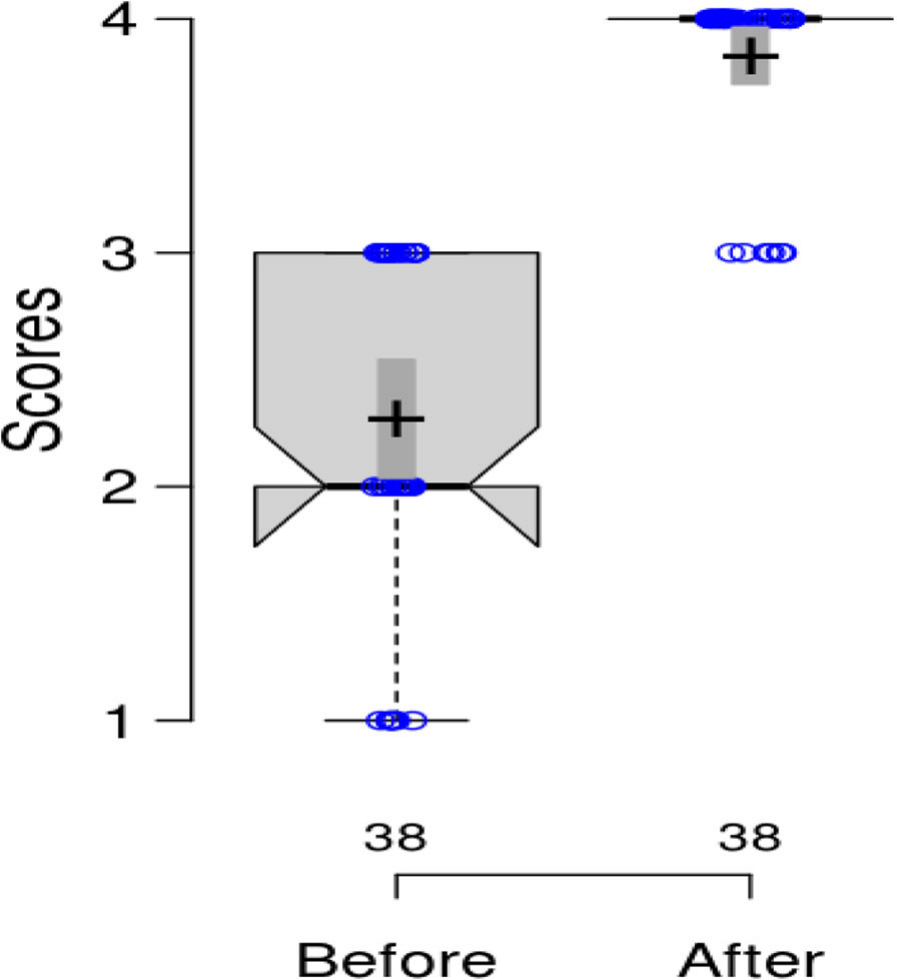
Boxplots with notches corresponding to the scores developed through the surgeon questionnaire scale before and after the application of the CRS technique

Surgeon’s impression assessed with the proposed questionnaire scale demonstrated that CRS leads to extremely high improvement in the stability of operative field (Fig. 1).

## Discussion

Retrograde Intrarenal surgery is being increasingly used in renal stones. This is partly due to advancements in surgical and endoscopic technology. Although RIRS is less technically challenging then PCNL, laser lithotripsy can be challenging. Respiratory renal movement and proper laser settings are the two main contributing components of successful lithotripsy [8]. In order to minimize kidney displacement due to respiratory motion, several methods have been previously suggested including high frequency ventilation [9], PA [3], abdominal compression [10], and general anesthesia with low ventilation [11]. There have been many modifications suggested, but each technique has its own advantages and limitations.

Öberg and Sjöstrand were first to describe the HFJV in 1967 [9]. Initially it was utilized in otolaryngology, bronchial and lung surgery [12]. In the past decade, an increasing interest in HFJV’s advantages in keeping target organs relatively motionless during surgery to optimize surgical precision has grown. In regards to stone treatment, in 1988 Warner and colleagues were the first to demonstrate the potential benefits of HFJV for shock wave lithotripsy (SWL) and since then several studies have confirmed the benefits of HFJV in SWL [4, 13, 14]. Popiolek et al. was the first to present high frequency positive pressure ventilation in RIRS during the 2017 World Congress of Endourology, demonstrating the true necessity of stabilization of the operative field [15]. However, while high frequency ventilation may facilitate quicker stone removal via the occurrence of less adverse stone movements, this method also makes it impossible to use inhalational anesthetics and does not monitor end-tidal CO_2_ levels nor the volume of exhaled air [5]. Therefore in our opinion, high frequency ventilation remains a suboptimal approach toward anesthesiological support during RIRS.

The periodic apnea technique has been presented as useful during RIRS to minimize adverse motion of target organs [3]. This method uses preoxygenation with FiO_2_ of 1.0, followed by a MV pause. The criterion for starting over the ventilation was the SpO_2_ drop below 93%. However, there is a possible delay between the true SpO_2_ drop and the pulse oximeter readings [16, 17]. Prolonged apnea leads to an inevitable hypercapnea which, in turn, results in adverse effects in patients with compromised cardiovascular status, increased intracranial pressure, metabolic acidosis or hyperkalemia [18]. The hypoxemia tolerance varies highly depending on the patient’s individual condition. SpO_2_ decrease is usually accompanied by a compensatory circulatory system response, which requires shorter ventilation pauses in aged patients, while younger patients can tolerate up to 6 min apnea [19].

Abdominal compression in the form of belts or plates was previously described in SWL. Belt compression in a group of 50 patients provided reduction of the kidney motion amplitude by an average of 32% detected by fluoroscopy control [20]. Plate compression in a group of 10 volunteers and 10 patients reduced kidney motion by 4 mm on average and increased SWL efficacy by 23% detected by ultrasound control [10]. Currently, no information regarding the applicability of abdominal compression in RIRS exists. The technique of general anesthesia with low ventilation (LV) was previously described by Kourmpetis et al. who suggested the following settings of GA during RIRS: respiratory rate ≤ 8/min and tidal volume < 500 ml [11]. According to the results of their study, LV was associated with better fragmentation, removal, and processing rates but not operating rate. Although end-tidal CO2 in the LV group was 50 mmHg this finding was not associated with clinical side effects. However, this level of end-tidal CO2 is defined as mild hypercapnia and may cause possible negative health consequences [21].

**Fig. 2:**
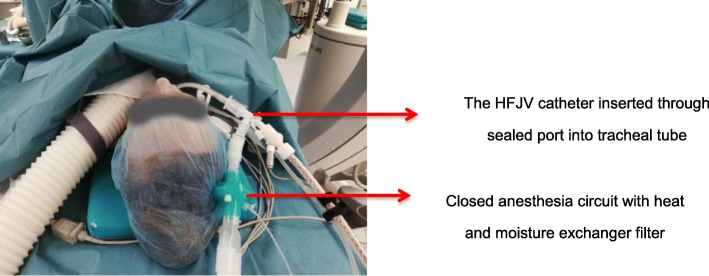
Tubing and connections in CRS

Our technique of combined two respiratory approaches, consisting of HFJV and SVMV, which thereby provided significantly better conditions for stone dusting assessed by expert urologists, all while precluding the development of hypercapnea. No CRS associated complications developed during or after the operation. In order to avoid any the following preventive measures were undertaken: the insufflation catheter was inserted through a sealed adapter into the tracheal tube thus, direct contact of the catheter with the trachea was excluded; development of tracheitis described in the literature is more attributed to the use of machines without gas conditioning option and the use of high pressure. We used “ZisLINEJV-100” machine with the air conditioning option and low working pressure of 0.3–0.6 bar; the heat and moisture exchanger filter was used in a closed respiratory circuit (Fig. 2).

Worth to mention disadvantages of CRS: expenditures associated with the cost of one insufflation catheter per patient; the necessity of purchasing HFJV machine; the inability to control the concentration of oxygen (FiO2) in some HFJV machines.

Our study has several limitations. First, our sample size was restricted to 38 patients. Despite this, we were able to demonstrate a statistically significant difference using the developed surgeon questionnaire scale; indicating that the use of CRS was beneficial. Secondly, our study lacks objective criteria for evaluation such as operating or fragmentation and dusting rates. However, we have deliberately simplified our study protocol due to the fact that variables such as fragmentation and dusting rates are inherently dependent on factors such as stone size, stone density, renal anatomy, the surgeon’s expertise, where the main factor being assessed in this study was operative field stability. Lastly, we didn’t corroborate our findings with the stone free rates of patients since we sought only to assess the feasibility and safety of reducing respiratory kidney motion and thus providing better conditions for intracorporeal lithotripsy. Due to the limitations of this pilot study, additional investigation will be needed to address the aforementioned concerns.

## Conclusions

Our impression is that according to the urologist assessment the novel combined respiratory approach consisting of HFJV and SVMV provides better conditions for stone dusting through reduced respiratory kidney motion and is not associated with adverse health effects or complications. Further larger sample studies are needed to confirm these findings and elucidate its effect on stone free rates.

## Data Availability

The datasets used and/or analyzed during the current study are available from the corresponding author on reasonable request.

## Abbreviations

CRS: Combined respiratory support
GA: General anesthesia
HFJV: High frequency jet ventilation
MV: Mechanical ventilation
PA: Periodic apnea
RIRS: Retrograde intrarenal surgery
SVMV: Small volume mechanical ventilation
SWL: Shockwave lithotripsy
